# Resilience in Adult Health Science Revisited—A Narrative Review Synthesis of Process-Oriented Approaches

**DOI:** 10.3389/fpsyg.2021.659395

**Published:** 2021-06-03

**Authors:** Nina Hiebel, Milena Rabe, Katja Maus, Frank Peusquens, Lukas Radbruch, Franziska Geiser

**Affiliations:** ^1^Department of Psychosomatic Medicine and Psychotherapy, University Hospital Bonn, Bonn, Germany; ^2^Department of Palliative Medicine, University Hospital Bonn, Bonn, Germany

**Keywords:** resilience, positive adaptation, mechanism, positive outcome, future directions, process-oriented approach

## Abstract

**Purpose:** This article aims to identify how the term “resilience” is addressed in adult health science due to ongoing criticism about the lack of consistency in its conceptualization.

**Method:** Two databases (PubMed and PsycArticles) were searched to retrieve reviews published from 2015 up until 2020 on the general conceptualization of resilience. All reviews had to meet specific inclusion criteria, which resulted in the inclusion of 18 articles. After discussing different conceptualizations regarding the process-oriented approach of resilience in adult health research, we will highlight some mechanisms that are supposed to be involved in the resilience process.

**Results:** Research on resilience in health sciences confronts three core difficulties: defining positive outcome for a processual construct, describing different trajectories within the process, and identifying mechanisms that mediate resilience.

**Conclusion:** The definition of resilience in mental health research as a multidimensional adaptation process is widely accepted, and multiple research paradigms have contributed to a better understanding of the concept. However, the definition of a processual construct in a way that allows for high expert consensus and a valid operationalization for empirical studies remains a challenge. Future research should focus on the assessment of multiple cross-domain outcomes and international and interdisciplinary prospective mixed-method longitudinal designs to fill in the missing links.

## Introduction

Over the last two decades, resilience has become an extremely popular topic in health science. It seems to be the conceptual shooting star whenever people need a positive adaptation approach to deal with complex, difficult, and even hopeless situations (Stainton et al., 2019).

Despite a proliferation of resilience research in mental health science, some concerns regarding the clarity and utility of the term are still unresolved (Luthar et al., 2000; Windle, 2011). Inconsistencies in the definition of resilience have led to varying meanings and concepts. This has prevented the further development and practical implementation of a unified recognized model of resilience and intervention strategies that derive from it (Angeler and Allen, 2016). When it comes to resilience, we seem to lack a comprehensive, meaningful way of measuring adaptability, and crisis (Panter-Brick and Leckman, 2013).

The objective of this article was to highlight how resilience is typically conceptualized in adult health research with a particular emphasis on process-orientated approaches. Specifically, we were interested in the most salient conceptual and methodological discrepancies within the process-orientated resilience discourse, and the challenges arising from them. Since there are already many existing reviews on the topic, we aimed to synthesize consistencies in reviews from 2015 to 2020 in adult health science, and highlight some prevalent attributions of resilience that we, in line with other authors, deem worthy of reconsideration. We will illustrate different global assumptions and perspectives that are considered controversial in mental health science Within resilience models, there is still a dearth of conceptualizations on mechanisms that mediate the effect of different variables on a resilient process or outcome. Therefore, we examine some mechanisms involved in the resilience process. We will conclude with some ways that future research could revisit to solve the conceptual difficulties linked to resilience and implications for clinical implications. Our article should thus be read as a narrative synthesis of some major methodological and conceptual problems related to resilience as a dynamic adaptation process. It is worth noting, however, that our article does not aim to dismiss or replace previous conceptions of resilience.

### Background

The concept of resilience in health research originated from developmental psychology (Werner and Smith, 1982; Garmezy, 1991) and emerged in four major waves (for a review, see Wright et al., 2013). In the first wave, the aim was merely to identify protective factors that contribute to individual resilience. Resilience was mostly a trait-oriented approach to detect a certain resilient personality type (Block and Block, 1980; Connor and Davidson, 2003; Hu et al., 2015). Many resilience scales (RS) used now in resilience research are still based upon this assumption [i.e., Resilience Scale (RS), Wagnild and Young, 1993]. Nowadays, there is a wide consensus that resilience is an adaptation process that goes beyond the construct of a personality trait (Leys et al., 2018; Hiebel et al., 2021), although some still consider it a useful approach (Hu et al., 2015). Instead, personality seems to be one of many potential protective factors (Luthar et al., 2000). In the second wave, resilience was embedded in developmental and ecological systems. The research focused more specifically on understanding resilience as a complex and systematic phenomenon arising from many processes (Cicchetti, 2010). From inception of the ongoing interactions, the third wave tried to create interventions and training to foster resilience when it was not likely to occur naturally. Lately, resilience research has focused in the fourth wave on multilevel dynamics linking genes, neurobiological aspects of development and adaptation, behavior, context, and their specific interplays (Cicchetti, 2010). At the same time, interdisciplinary perspectives on resilience are coming together (Wright et al., 2013).

To sum up, over the last 40 years, the concept of resilience has taken multiple meanings in different contexts, changing from a trait-oriented to an outcome- or process-oriented approach (see Status Quo and challenges in defining resilience). Although a strong knowledge base has been revealed and resilience is defined as a dynamic multidimensional construct (Masten and Cicchetti, 2016; Malhi et al., 2019), there is much we do not know (Wright et al., 2013; Malhi et al., 2019). Next, we take a closer look at the status quo and challenges to defining and assessing resilience.

#### Status Quo and Challenges in Defining Resilience

Resilience is conceptualized in various possible meanings in the mental health literature and the wider resilience research (Windle, 2011; Richter and Korsch, 2017; Ayed et al., 2019). For instance, Windle (2011) published a concept analysis of how resilience could be defined. She highlighted the inconsistencies in the use of the term relating to the nature of potential risk (i.e., being exposed to an isolated stress event or persisting stress over life span) and protective processes (i.e., on an individual or environmental level) across multiple disciplines such as psychology, ecology, biology, and psychiatry. Although there is no universal definition, different common themes in how researchers used the term “resilience” in the context of their studies were identified (Aburn et al., 2016; Rudzinski et al., 2017). The most commonly cited process-oriented definition considers resilience to be patterns of positive adaptation despite significant adversity (Rutter, 2012). Another definition highlights resilience as the ability to bounce back and recover from adversity (Garmezy, 1991; Kumpfer, 1999; Edward et al., 2009). In a different instance, resilience is studied in relationship to specific outcomes such as the presence or absence of good mental/physical health or illness (Kalisch et al., 2015). In both process- and outcome-based approaches, exposure to significant adverse events is a central requirement for resilience. From this perspective, resilience can only be identified if the individual has been exposed to risk. Different internal, external, and environmental factors, including the role of (epi)genetics, personality traits (e.g., hardiness), cognitions (e.g., goal-orientation, self-efficacy), and social, socioeconomic, and cultural resources have been studied in relationship to resilience (Malhi et al., 2019). While the outcome- and process-oriented approaches greatly overlap, they differ in quality (Ayed et al., 2019).

Lately, resilience is increasingly considered in a broader, dynamic multifaceted process approach, including a system perspective (Masten and Cicchetti, 2016; Masten and Motti-Stefanidi, 2020). The goal is to present a more unified approach to research on an individual, family, and community level. Here, temporal aspects of resilience trajectories (e.g., pre-adversity functioning, acute vs. chronic adversity; Bonanno et al., 2015), contextual (resilience changes in different circumstances), and individual and social processes are thought to affect resilience. On the individual level, factors range from (epi)genetics and neurobiological to cognitive, emotional, and behavioral aspects. As social components, family, friends, colleagues, or even more distal macro-systems (e.g., government) are taken into account. As such, resilience can develop and/or be spread over time by multiple processes.

One theory which reflects the changing understanding of resilience as integrative and multifaceted is the multi-system model of resilience (Liu et al., 2020). The model maps different sources of resilience capacity through three systems: an intra-individual source (gender, sex, biology, physiology, health behavior), an interpersonal source of resilience (education, family, competence and knowledge, interpersonal relationships and social groups, skills, and experience), and socio-ecological sources of resilience (access, formal, and informal institutions, geography, socio-economic status). All these resources could be mobilized in face of adversity, risk exposure, and potential challenges. At the same time, they are supposed to operate in the background in order to sustain wellbeing.

The conceptual diversity in defining resilience has also been evident when it is studied in acute and chronically stressful life events, in divergent populations ranging from children to the elderly, from families to larger neighborhoods and communities, and in adversities ranging from the daily hassles of arguments with family members and work difficulties to more acute life events like bereavement, unemployment, and even chronic events (Bonanno and Diminich, 2013; Infurna, 2020).

To sum up, most definitions of resilience are based on three core qualifiers: adversity, positive adaptation, and positive outcome (Kunzler et al., 2018). Although the conceptualization of resilience has gained some stability in regard of a dynamic multidimensional process-oriented approach over the last two decades, this does not mean that the persistent debate around this concept is obsolete (Stainton et al., 2019).

#### Current States and Challenges for the Assessment of Resilience

Inconsistencies in the theoretical clarity of resilience negatively impacts the operationalization of the construct. We still lack a gold standard for the assessment of resilience (Windle et al., 2011). Instead, we often estimate resilience using self-report RSs or measures of surrogate outcomes such as mental health constructs (i.e., the presence or absence of physical/mental well-being or disorders/illness), functionality (i.e., employment, marriage, good interpersonal relations), or stress perception (Cosco et al., 2017; Chmitorz et al., 2018). As far as we know, there is no universally established outcome measure for resilience itself.

Resilience scales typically measure it as comparable to a stable personality type (i.e., RS, Wagnild and Young, 1993) or assess a few constructs (i.e., the availability of internal and external resources) that elicit behavior or attitudes linked to resilience (i.e., self-efficacy, social support) or to maintain mental health in response to adversity [i.e., Connor–Davidson Resilience Scale (CD-RISC), Connor and Davidson, 2003]. Often, there is no clear distinction as to whether these concepts are viewed as a resilience factor (i.e., a variable predicting or promoting adaptation) or a resilience outcome (i.e., an indicator of the level of adaptation reached). This probably reflects the circular nature of the resilience process whereby successful adaptation enhances the ability and confidence to cope with further crisis. As a result, most RSs focus on proactive cognitive and behavioral strategies to cope with the negative impacts of adversity. For instance, common items in RSs include ego strength, goal orientation, optimism, and adaptive coping associated with an individual's agency (i.e., “I usually manage one way or other,” RS, Wagnild and Young, 1993), whereas negative emotional responses are primarily framed as inappropriate or harmful (i.e., “I can manage my emotions,” Washington Resilience Scale, Ahn, 1991; “I can handle unpleasant feelings,” CD-RISC, Connor and Davidson, 2003). If, however, we include the human experience of being affected by crisis and struggling through different stages, then non-agency experiences such as desperation or recognizing one's own helplessness may also be seen as valid, even necessary, aspects of a dynamic resilience concept (Richter and Korsch, 2017; Infurna, 2020).

Although contemporary definitions of resilience highlight the process of adaptation rather than the trait approach, to our knowledge, only one RS assesses it in a dynamic way as the ability to bounce back from adversity (i.e., “It does not take me long to recover from a stressful event,” The Brief Resilience Scale, Smith et al., 2008). In military science, the Deployment Risk and Resilience Inventory (DRRI-2; Vogt et al., 2013) measures (pre-)deployment-related risks (i.e., prior stressors, perceived stress, family stressors) and resilience factors (i.e., support from family and friends) in service members and veterans and addresses not only protective factors but also effects of (pre-)adversity itself. Lately in developmental science, the invention of a rapid battery for risk/resilience assessment that takes a broad perspective of risk and resilience by assessing seven factors that span intrapersonal (i.e., self-reliance, emotion dysregulation), interpersonal (i.e., positive/negative relationships), and wide-ranging external contexts (i.e., stressful events) has taken place (Moore et al., 2020).

Moreover, independent predictors such as sociocultural variables are not assessed in RSs (Pangallo et al., 2015), thereby ignoring their cumulative influence on resilience. For instance, Bonanno et al. (2007) showed that the prevalence of resilience was predominantly predicted by the participant's gender, age, race/ethnicity, education, level of trauma exposure, income change, social support, frequency of chronic disease, and recent and past life stressors.

Thus, RSs can only provide a selective estimation of contributing factors rather than a valid assessment of the construct. These inconsistencies warrant further development of resilience measurements.

### Aims of This Review

In this review, we aimed to (a) critically evaluate and discuss how process-orientated approach defined by the resilience construct is typically conceptualized in adult health research with regard to the adaptation criteria and the adaptation process and to (b) conclude with a set of suggestions for further studies and clinical implications based on the extracted results.

## Methods

To do so, we combined a systematic literature review in accordance with the Preferred Reporting Items for Systematic Reviews and Meta-analyses (PRISMA) guidelines (Moher et al., 2015), with a narrative synthesis based on the guidelines of Evans (2002). Due to the large number of high-quality peer-reviewed reviews, our systematic literature review comprises secondary research reviews. To our knowledge, this is the first article that synthesizes the information which were included in reviews published from 2015 to 2020 on resilience and in particular process-oriented approaches.

### Literature Search

Searches were conducted on 27^th^ of December 2020 and limited to two major health-related databases: PubMed and APA PsycArticles. The target population were adults (18+). The phenomena of interest resilience and the context of search (systematic) reviews. We searched databases using the term “resilien^*^” from the title, including the following terms: “resilient,” “resilience,” and “resiliency.” We used these general search term to capture the overall understanding of the term “resilience” without going into the discussion of resilience in different specific groups such as children, families, or patients. The following limiters were placed if possible for searches: peer-reviewed, review, reference available, publication date between 2015 and 2020, and English or German language.

**Figure 1:**
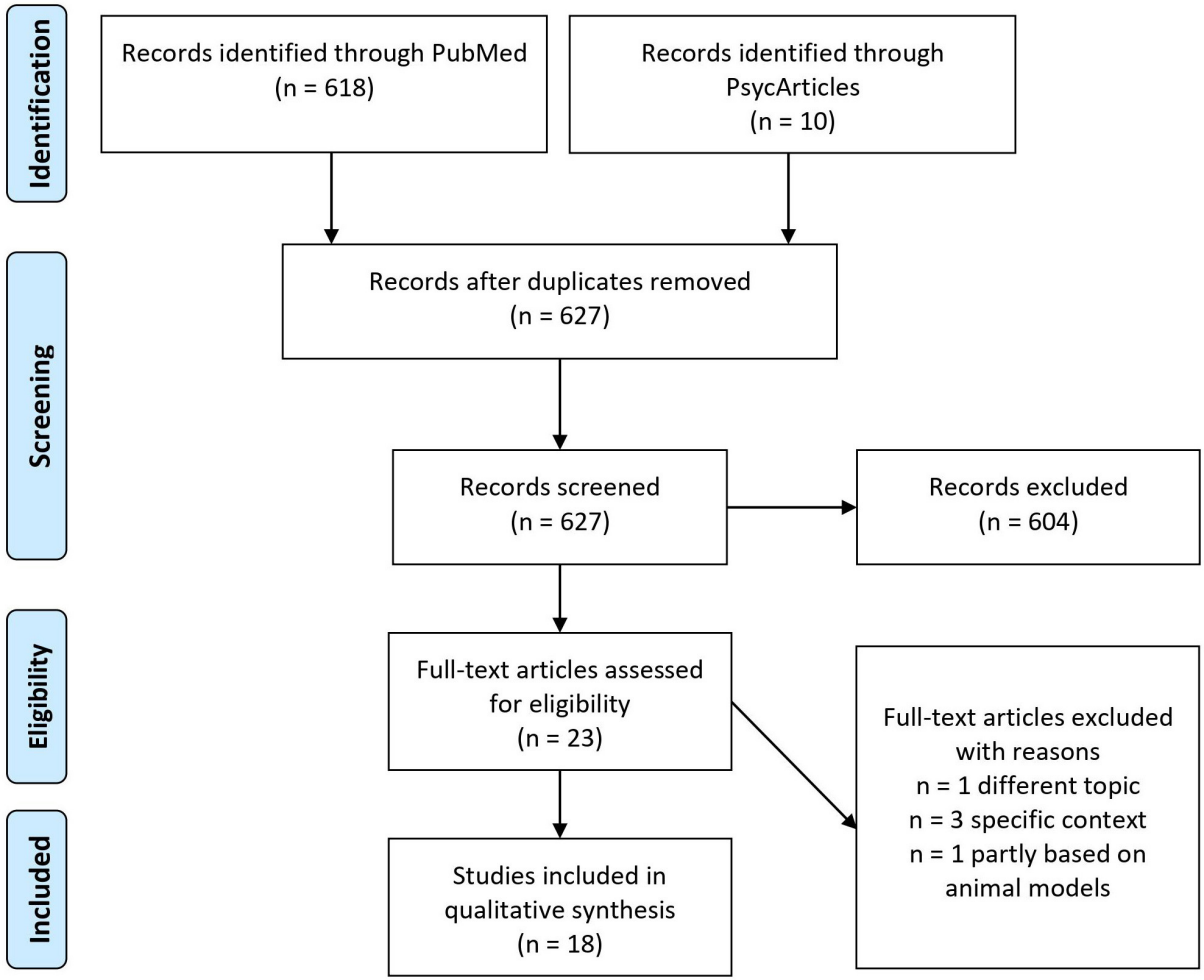
PRISMA search strategy.

All references were exported into Citavi version 6.8.0.0, and duplicates were removed. The titles and abstracts of the texts were screened. Following this, the full texts were examined. Figure 1 illustrates our search strategy based on the PRISMA procedure.

### Eligibility Criteria

The inclusion and exclusion criteria are displayed in Table 1. Review articles were included if they met the following criteria: (1) population include adults (18+), (2) focus solely on individuals in health science, (3) time period 2015–2020, (4) publication criteria: written in English or German language, peer-reviewed, reviews, and (5) include a clear conceptualization of resilience and/or process-oriented resilience approach. Criteria one led to the exclusion if the review only focused on child's and adolescents. Based on criteria two we excluded articles focusing on resilience at a group-level like families, society, or government. Also specific contexts like sports, disasters, etc. were excluded. Criteria three was set since the included reviews already reach back in time, we only focused on reviews published in the last 5 years. Criteria four was extended including narrative, systematic, and literature reviews. Criteria five was understood as an explicit definition or concept of resilience as adaptation process or bouncing back or referring to mechanism in general including frameworks. This reflects the wider decision to capture how process-oriented approaches are typically defined. Thus, reviews solely focusing on interventions or scale development or evaluation were excluded.

**Table 1:** Inclusion and exclusion criteria based on PICo.

**Inclusion criteria**	**Exclusion criteria**
1. Population: adults (18+)	1. Population: minor 18
2. Context: individuals, health science	2. Context: groups (i.e., family, social, or political systems, specific patient group) specific contexts (i.e., sports, environmental, developmental, disasters, animal)
3. Time period: 2015–2020	3. Time period: before 2015
4. Publication criteria: English or German language; peer-reviewed, reviews, systematic reviews, literature review	4. Publication criteria: full-text not available, empirical study, gray literature, books
5. Phenomena of interest: resilience, process-oriented approach, frameworks	5. Phenomena of interest: main focus intervention, scale development/evaluation

### Data Analysis

The narrative synthesis process was based on Evans (2002). We identified the key findings of each review and related the identified themes across reviews. Finally, through an iterative process of continuous categorization into areas of similarity, critical reflection, reference to the original data, and feedback, the final themes were agreed upon and specified.

## Results

### Screening and Selection

The initial search yielded 628 articles. One duplicate had to be removed. Of the remaining articles, 604 were removed after titles and abstracts were examined. The remaining 23 articles were then assessed for eligibility. Eigtheen articles were included in the qualitative synthesis (Figure 1). The main qualitative results of data analysis will be presented in Table 2. Regarding the conceptualization of resilience as a process-based approach we extracted three major themes: the adaptation criteria, resilient trajectories, and resilient mechanism. Next, the results will be discussed.

**Table 2:** Characteristics of the included reviews and extracted themes.

**References**	**Title**	**Method**	**Search period**	**Focus**	**Extracted themes**
(1) Aburn et al., 2016	What is resilience? An integrative review of the empirical literature	Integrative review	2000–2013	**Conceptualization of resilience in common themes**	*Conceptualizations* (1) **No universal definition** (2) Key resilience researchers (3) **Resilience as a contextual and dynamic process** (4) Rising above to overcome adversity (5) **Adaptation and adjustment** (6) Ordinary magic (7) **Good mental health as a proxy of resilience** (8) **Ability to bounce back**
(2) Ayed et al., 2019	Conceptualizing resilience in adult mental health literature: a systematic review and narrative synthesis	Systematic review	1974–2016 1806–2016 1946–2016	**Conceptualization of resilience in common themes Processes** Personal and social resources	*Conceptualizations* (1) **Resilience concepts Process (immunity, bouncing back, growth)** (2) Personal resources (traits, talents, skills, interests) Social resources (surrounding environment and social context)
(3) Bonanno et al., 2015	The temporal elements of psychological resilience: an integrative framework for the study of individuals, families, and communities	Integrative framework review	/	**Temporal elements of resilience** Crisis **Trajectories Resilient outcomes** (Families, Communities)	(1)Acute and chronic events (2) Levels of exposure (3) Proximal and distal exposure (4) **Baseline adjustment and resilient outcome** (5) **The limits of diagnoses and the problem of averages (absence of psychopathology is not resilience)** (6) **Trajectories of positive adjustment**
(4) Chmitorz et al., 2018	Intervention studies to foster resilience—a systematic review and proposal for a resilience framework in future intervention studies	Systematic reviews on randomized controlled trials	1979–2014	**Definition of resilience Assessment of resilience** (Intervention studies)	(1)**Heterogeneous definitions** (2) **Resilience scales** (3) **Surrogate outcome measures (resilience factors, mental health related constructs, stress reduction)** (4) Intervention studies
(5) Cosco et al., 2017	Operationalising resilience in longitudinal studies: a systematic review of methodological approaches	Systematic reviews	/	**Operationalization methods**	(1)Adversity (2) **Positive adaptation criteria (i.e., mental health, distress)**
(6) Darling Rasmussen et al., 2019	Attachment as a core feature of resilience: a systematic review and meta-analysis	Systematic review and meta-analysis	/	**Attachment as resilience mechanism**	*Mechanism* (1) **Attachment is a pre-requisite of positive adaptation**
(7) Infurna, 2020	What does resilience signify? An evaluation of concepts and directions for future research	Review	/	**Conceptualization of resilience Future directions**	*Conceptualization* (1) **Defining resilience is heterogeneous** (2) **Individualized** (3) **Phenomenon resilience is multidimensional** (4) **Public narrative** *Future directions* (1) **Concept of anticipation** (2) **Prospective longitudinal designs that incorporate mixed-methodology**, (3) **Multidimensional assessments** (4) **The examination of multiple adversities that transpire in relatively close proximity to one another**
(8) Infurna and Luthar, 2018	Re-evaluating the notion that resilience is commonplace: a review and distillation of directions for future research, practice, and policy	Review	/	**Definitions and assessments** Past resilience research **Future directions**	*Definitions and assessments* (1) **Controversy in defining and assessing resilience** *Future directions*(2) Replicating trajectories across samples and outcomes
					(3)**Illuminating the processes underlying resilience and** (4) **Incorporating a multidimensional approach**
(9) Kalisch et al., 2015	A conceptual framework for the neurobiological study of resilience	Review	/	**Cognitive appraisal as general resilience mechanism** Positive appraisal style theory of resilience	*Mechanism* (1) **Positive appraisal is a general resilience mechanism**
(10) Kunzler et al., 2018	Aktuelle konzepte der resilienzforschung	Analysis and discussion of current works and expert recommendations	/	**Assessment and operationalization** Protective factors **Mechanism Future directions**	(1)**Defining resilience is heterogeneous** (2) **Resilience is dynamic and adaptable** (3) **Resilience factors** *Mechanisms* (4) **Positive appraisal** *Future directions* (5) **Multicenter design** (6) **Longitudinal design** (7) **Calculating resilience scores to measure resilience**
(11) Kleim and Kalisch, 2018	Wer bleibt gesund? zum problem der vorhersage von resilienz	Analysis and summary of recent studies on psychosocial and neurobiological resilience predictors	/	Protective factors **Future directions**	(1) **Resilience as dynamic adaptation** (2) **Resilient trajectories** (3) Psychosocial predictors of resilience (4) Neurobiological predictors of resilience *Future directions* (5) **Prospective multicenter research designs** Computer modeling
(12) Lindert et al., 2018	Verläufe von resilienz – beispiele aus längsschnittstudien	Qualitative literature review	/	Age-related trajectories	(1)**Defining resilience is heterogeneous** (2) **Adaptation processes can only be assessed longitudinal** (3) **International and interdisciplinary cooperation's within cohort studies**
(13) Liu et al., 2018	Biological and psychological perspectives of resilience: is it possible to improve stress resistance?	Review	/	**Neurobiological mechanism** Intervention	*Mechanism* (4) **Medial pre-frontal cortex** (5) **Hippocampal pathways** (6) VTA-NAc pathways intervention (7) Altering neural activity (8) Neuropharmacological approaches (i.e., ketamine, NPY)
(14) Daly, 2020	Resilience: an integrated review	Systematic review	2013–2018	**Mechanism**	*Mechanism* (1) **Hardiness strengthens the ability to harness resources** (2) **Regulatory flexibility fosters positive functioning** (3) **Challenges enhance the ability to rebound**
(15) Pangallo et al., 2015	Resilience through the lens of interactionism: a systematic review	Systematic reviews	All years	Assessment and operationalization of resilience Quality of assessment Future research	*Assessment and operationalization of resilience* (1) **Despite the range of conceptual approaches (trait, process, outcome), there is little variation in the scope of the instruments being assessed** (2) **Consistent use of characteristics** (3) **Absence of sociocontextual and demographic predictors** *Quality of assessment* (4) Theory formulation (5) Internal validity (6) Psychometric evaluation
					*Future direction* (7) Clarifying context between chronic and acute stressors (8) **Definition core antecedents (adversity) and consequences (positive adaptation)**
(16) Sisto et al., 2019	Toward a transversal definition of psychological resilience: a literature review	Systematic reviews	2002–2019	Conceptualization of resilience in common themes	*Conceptualization* (1) **Ability to recover** (2) **Type of functioning that characterizes the individual** (3) **Capacity to bounce back** (4) **Dynamic process evolving over time** (5) **Positive adaptation to life conditions**
(17) Stainton et al., 2019	Resilience as a multimodal dynamic process	Narrative review synthesis	/	Multimodal dynamic process Protective factors Mechanisms	*Conceptualization* (1) **Defining resilience is heterogeneous** (2) **Resilience as a dynamic process** *Protective factors* (3) Psychological and social factors(4) Neurobiological protective factors (5) Genetic protective factors (6) Neurocognitive protective factors *Mechanisms* (1) **Positive reappraisal** (2) **Differential-susceptibility hypothesis (genetics)** (3) **Steeling effect**
(18) Yao and Hsieh, 2019	Neurocognitive mechanism of human resilience: a conceptual framework and empirical review	Framework	/	Resilience as a dynamic process Mechanism	*Mechanisms* (1) **Cognitive appraisal of adversity regarding stressor perception, severity and** (2) **the role of cognitive appraisal in cognitive-emotional processes** (3) **Pre-frontal cortex as important contributor to resilience**

*Extracted themes the narrative synthesis is based on are typed in bold*.

## Discussion

### Conceptualization of Resilience

Based on the narrative synthesis of multiple reviews, and in line with several authors, we identified some conceptual difficulties around resilience as worth discussing. We mainly focus on the core components of a process-based approach—the adaptation criteria and the adaptation process itself. We begin by outlining different markers in health science that describe whether the outcome is “resilient.” We then take a closer look at different resilience trajectories associated with the process-oriented approach before we highlight some proposed mechanisms underlying a positive adaptation. We end by drawing some conclusions for future research.

#### The Adaptation Criteria: Functionality and Health as Markers of Positive Outcome

Some of the current challenges in the resilience field in adult mental health science might be determined by the criteria researchers define as a resilient or good outcome (Yao and Hsieh, 2019). Despite the multidimensional nature of resilience, it is primarily operationalized dichotomously (Kunzler et al., 2018). As stated, most research designs estimate resilience by the presence or absence of traditional markers of positive outcomes such as physical/psychological health/illness/disorder, functionality (employment, marriage, good interpersonal relationships), or stress perception (Cosco et al., 2017; Chmitorz et al., 2018). The use of such a categorical classification system (healthy vs. ill; functional vs. dysfunctional) in the operationalization of resilience, however, poses several problems. Firstly, the absence of a physical/psychological disorder is not a resilient outcome *per se* (Bonanno et al., 2015). Since psychological phenomena are not binary (for instance, a person is happy or not), quantifications of resilience based on a dichotomous classification system seem rather inappropriate (Kunzler et al., 2018). Secondly, the dichotomy of the surrogate outcome measure does not allow comorbidity between several disorders, which, however, occurs quite commonly. Such symptom overlaps do show a great impact on mental health constructs, but as far as we know, they are not taken into consideration in empirical studies on resilience. For instance, more than half the people suffering from addiction are affected by multiple addictions (polytoxicomania) and additional somatic (i.e., damage of liver or lungs, inflammation of the pancreas) and/or psychological disorders (e.g., depression, anxiety disorder, psychotic disorder; Ross and Peselow, 2012; Infodrog, 2015). Despite being abstinent, an individual might still suffer from somatic or psychological disorders. Would this person then be resilient because of abstinence from alcohol or substance abuse, since the person is still mentally/physically impaired? Such heterogeneity in a resilient outcome is often identified in research (Bonanno, 2004). Even those individuals diagnosed with psychiatric disorders experience phases with fewer symptoms or behavioral difficulties or with increased quality of life or experience of meaning despite the presence of symptoms. A single category of (non-)psychopathology does not allow accounting for changes within that category. Its focus is simply too narrow (Bonanno et al., 2015).

Furthermore, being healthy or ill is not as easy to distinguish based on a simple threshold level (Kunzler et al., 2018). From this perspective, we cannot draw on categories like sick or healthy as unique markers for a resilient outcome. Illness and health are not a continuum, but two independent phenomena (“Health is a state of complete physical, mental and social well-being and not merely the absence of disease or infirmity,” Constitution of the World Health Organization, 2021). We should see those terms more as different expressions on the respective dimension, being less sick or healthier, respectively. Kalisch et al. (2015) lately suggested a different approach to quantify whether an outcome is good (see also Kunzler et al., 2018). They proposed a quantification of a global score for resilience based on a sum or mean score of multiple variables differing in their functional dimensions (depression, anxiety, somatic dysfunction, behavioral, cognitive resilience mechanisms). Changes in dysfunction could then be estimated even if the threshold between healthy and sick has not been bypassed. Recently, Infurna and Luthar (2017) composited such a total score across five different outcomes. They used three indicators of subjective well-being in life (satisfaction, negative affect, and positive affect), two indicators of health (subjective perceptions of general health and physical functioning), and several protective factors (anticipating reliable comfort when distressed, maintaining social connectedness, and engagement in everyday role activities, along with socio-demographic indices of age, gender, and education) that might promote resilience. In contrast to life satisfaction, whereby 66% of participants were likely to be resilient, only 26 and 19% were classified as resilient for positive affect (26%), negative affect (19%), general health (37%), and physical functioning (29%). Each of the five outcomes showed heterogeneity in degree and rates of change. For some, it took less time to return to their previous level (i.e., of life satisfaction), whereas others did not fully reach their pre-adversity level at all. When they considered all five indicators simultaneously, only 8% showed “multidimensional” resilience, whereas 20% showed a non-resilient trajectory across all five indicators. Thus, there is not a unique surrogate of resilience that can be overlaid across all individuals and contexts. Whether an individual is classified as resilient strongly depends on the outcome chosen by the scientist who designs the study.

Closely related to the question of outcome heterogeneity is the reliance on average-level data on adaptation that are supposed to reveal basic descriptive information and longitudinal trends. However, such approaches have also been criticized for masking other interesting effects or even misread data. Since adaptation to adversity is often considered atypical, those limitations are especially relevant for resilience research (Bonanno et al., 2015).

A core principle of resilience as a dynamic adaptation process is its expected fluctuations across time and situations (Luthar et al., 2000; Rutter, 2012; Stainton et al., 2019; Masten and Motti-Stefanidi, 2020). Even if an individual demonstrates resilience in similar domains, we cannot expect him/her to be resilient in every context and at all times. For instance, an individual might adapt functionally to adverse events in the academic field (i.e., being unemployed) and struggle with the daily hassles of familiar conflict. Therefore, adaptation criteria need to take into account parallel processes in different areas of daily life (Luthar et al., 2000; Stainton et al., 2019). This leads to debates concerning *when* to access a resilient outcome (Wright et al., 2013). If resilience fluctuates over time, it seems quite arbitrary to estimate it within a single time point measurement. Also, after an adverse event, there are not only “proximal” (i.e., healthy adjustment) but also “distal” outcomes, further challenges in the personal environment that occur with a delay as a result of the events (i.e., economic consequences; Bonanno et al., 2015). This also contrasts with a fixed definition of an endpoint. One possible approach is therefore to define adaptation not by measuring an outcome at a fixed time point, but by defining resilient trajectories (Bonanno et al., 2015). We will come back to this point.

Finally, besides the personal perspective, the role of environmental resources and social selection for resilience (i.e., social, material resources), the access to those resources and the stability of the resources were highlighted (Chmitorz et al., 2018; Infurna, 2020). Based on their environmental and social backgrounds, individuals might be in a position that makes them more or less vulnerable in the face of adversity. For instance, resilience generally implies the ability to make good use of resources when confronted with adversity (Southwick et al., 2011). This implies that the resources needed to overcome resilience are also available in a given moment (Infurna and Luthar, 2018). However, a person can only navigate these resources if they are provided by the social context (Ungar, 2008). For instance, individuals with a good socioeconomic status living in a rich and stable context are much more likely to be resilient in adverse events compared to those living in a less favorable environment (Chmitorz et al., 2018).

#### Different Conceptualization in Resilient Trajectories

In the literature, different variations in a resilient trajectory have been observed across studies. Lately, pre-adversity levels of functioning (i.e., baseline mental or physical health, social support) and differences in the context of the adverse event (acute vs. chronic) are also taken into account. Whereas, acute adversity has been shown, in the majority of cases, to be followed by a “resilient” trajectory with “immunity/resistance” to dysfunction or a minimum impact on dysfunction, being exposed to chronic adversity fosters more disruption in functionality than “bouncing back” would propose (Bonanno et al., 2015). The three most common resilient trajectories will now be briefly illustrated and discussed.

##### Immunity, Stability, or Resistance

One notion of resilience is a sense of immunity, stability, or resistance against adversity (Ayed et al., 2019). In line with this concept, the ideal trajectory of resilient adaptation in a crisis is seen as smooth and steady, with the permanent maintenance of functionality and mental health (Bonanno, 2004; Kalisch et al., 2015). While focusing on positive outcomes was important to overturn many deficit-focused models in the first wave of resilience research, the image of an “invulnerable” resilient individual (Werner and Smith, 1982) who “withstands” adversities (Mizuno et al., 2016) and/or maintains “well-being” is still present in the discourse of adult health science (Freund and Staudinger, 2015). Although this assumption may be true for some resilient trajectories (i.e., in acute crisis), it has been critiqued for its narrow focus (i.e., Stainton et al., 2019; Infurna, 2020). It seems extremely unlikely that individuals would remain completely unaffected, at least in the face of chronic adversity (Bonanno et al., 2015). Furthermore, if an adverse event is too overwhelming, the adaptation process is very likely, at some point, to reach its limit, and any individual might experience symptoms of disorders (Stainton et al., 2019). It seems questionable whether this would necessarily qualify as being non-resilient.

Emphasizing such an ideal of lasting stability in the face of adversity in scientific research does impact the public discourse of resilience significantly. Labeling resilience as immunity might even stigmatize those who experience difficulties in facing adversity. It implies that an individual is supposed to be unaffected despite being confronted with significant adversity. If adjustment “takes too long,” then something must be wrong with the individual. However, this does not reflect the reality of human experience. In fact, cultural narratives of adjustment might even be disregarded (Infurna, 2020). For instance, when dealing with grief, time does not always heal all wounds. Individuals might still suffer, although they seem to function well on the outside (Devine, 2018). We should be careful not to turn resilience into a normative attribute and consequently apportion blame to those who display dysfunctionality in the face of adversity. Although dysfunctionality may be an indicator of a failed adaptation mechanism, it may also, on the contrary, be a sign of an ongoing and necessary process of dealing with challenges and injuries or losses. In fact, in biology, immunity does not imply any illness reaction to an antigen at all, but rather an early and attenuated defensive response. In the public discourse, however, an idealization of the concept of immunity or resistance as the most resilient trajectory risks to introduce a normative ideal which might be impossible for some individuals to accomplish and which may not even be desirable in some contexts (Infurna, 2020). Moreover, being dysfunctional for a certain time is not completely positive or negative, but rather a question of degree. As argued, resilience is not the same as the absence of psychopathology (Bonanno et al., 2015). Even those individuals with significant psychopathology or illness experience phases with fewer symptoms or behavioral difficulties or with increased quality of life or experience of meaning. Indeed, it has been proposed that resilient and vulnerable phenotypes can be present within the same disorder (Zannas and West, 2014; Malhi et al., 2019). Individuals might even experience personal gains due to dysfunctionality such as avoiding situations that are perceived as unpleasant (primary morbid gain) or receiving more attention from a loved one (secondary morbid gain), thus rendering the dysfunctionality partially functional. Thus, even those without psychopathology are not necessarily resilient (Malhi et al., 2019). On the other hand, some authors even propose reconsidering whether events or situations that show no negative impact upon an individual at all can still be defined as adverse (Ayed et al., 2019). From this perspective, individual trajectories that show immunity to adversity could not be considered resilient anymore. In fact, stability in the face of adversity has been shown to be a methodological and statistical artifact (Infurna and Luthar, 2018).

Moreover, the absence of a complete stress response in the face of adversity seems to be rather rare (Layne et al., 2007; Bonanno and Diminich, 2013). The majority of people mainly experience some disruption during or immediately after the event. The crucial point is, however, that the impact is mild and typically does not impair the normal level of functioning (Bonanno, 2004). Bonanno and colleagues (Bonanno and Diminich, 2013; Bonanno et al., 2015) referred to this trajectory as “minimal impact resilience,” which is supposed to reflect a stable adjustment within the framework of acute adversity.

##### Bouncing Back or Recovery

“Bouncing back” or “springing back” from adversity is another important conceptualization that describes a resilient trajectory (Smith et al., 2008; Edward et al., 2009; Bonanno et al., 2015; Masten and Cicchetti, 2016; Ayed et al., 2019). It implies that an individual can be affected negatively by adversity for a period of time and this can cause temporal disturbances (Kalisch et al., 2015). After an initial decline in function, the person returns to his/her more or less original pre-adverse level of functioning. That is why some researchers refer to this process as bouncing back to homeostasis (Tusaie and Dyer, 2004). The term “bouncing back” originated in material sciences and describes the ability of a material to return to its original shape after mechanical impact. For instance, a tennis ball bounces back because at the moment it hits the ground (i.e., stressor present), it is contorted into an oval shape. After the tennis ball bounces back, it regains its original shape. Thus, the deformation is a necessary ability to overcome the stressor. This conceptualization relates to recovery from illness when individuals regain a full and satisfying life after experiencing one or more episodes of mental illness (Stotland et al., 2008). Developmental science also refers to this trajectory as “breakdown and recovery” (Masten and Cicchetti, 2016). Bouncing back describes the extent to which a person returns to a normal state *after* the stressor is removed (Tarter and Vanyukov, 1999). Others argue that recovery should only be considered a resilient trajectory if it occurs during chronic adversity (“emergent resiliency,” Bonanno and Diminich, 2013; Bonanno et al., 2015). Lately, it was also found to be the modal response in the face of adversity (Infurna and Luthar, 2018).

##### Growth

Some researchers suggest that resilience contributes to an individual's growth after experiencing adversity (Tusaie and Dyer, 2004; Ayed et al., 2019). Unlike bouncing back, in which the individual returns to a pre-adversity level of functioning and adjustment, growth implies that the resilient person grows stronger and functions above the previous level. In the context of mental health, this conceptualization of resilience suggests that an individual might develop distress or psychopathological symptoms but learns to grow and responds more efficiently to similar challenges in the future (Meesters, 2014). This process is closely related to the concept of “post-traumatic growth” (Fooken, 2009), which is well-established in trauma and psycho-oncology research. Since both concepts show similarities with respect to an increased a level of functioning above the pre-adverse level, a clear conceptual distinction is not always made (Fooken, 2009).

#### Differences in the Mechanism or Interaction That Foster a Positive Adaptation

Recently, instead of confining themselves to simple associations between risk factors and outcomes, researchers have increasingly focused on the ongoing processes involved in the dynamic interaction between adversity and the affected person (i.e., the resilience mechanisms, as opposed to resilience factors). Those mechanisms refer to a catalytic effect by which a protective factor can change the influence of some risk factors (Rutter, 1993). In relationship to resilience, three different models—compensatory, protective, and challenge—have been proposed to explain how a protective factor (internal or external) may affect adversity positively (Fergus and Zimmerman, 2005; Bartley et al., 2010). In the compensatory model, the protective factor and risk factor impact an outcome independently, whereas in the protective model, resources or assets moderate or reduce the effects of adversity on an outcome. The latter might be accomplished by neutralizing or diminishing the effects of risk or by enhancing the effects of another positive factor. One mechanism for which there is extensive evidence is cognitive (re)appraisal (Gross and John, 2003; Kalisch et al., 2015), whereby an individual reframes an emotional response by changing the underlying cognitions that contribute to negative emotions. A third model of resilience is the challenge model, which proposes a curvilinear relationship between risk factor and outcome. The model suggests a negative outcome for low and high levels of risk and a positive outcome for moderate levels of adversity, whereby coping strategies or protective factors can be applied or learned most effectively. The idea is that, during their lives, adults are exposed to enough stress that they learn how to overcome it. When faced with future adversities, someone can draw upon their prior experiences and utilize those strategies that have been successful in the past (see also Daly, 2020). This mechanism is sometimes called an “inoculating” or “steeling effect” (Rutter, 2012). Surprisingly, there is still a lot of uncharted territory regarding resilience mechanisms. As far as we know, there is no consensus in science about whether there are only a few general mechanisms. The fact that resilience mechanisms are poorly understood is also reflected by the degree to which these reviews focus on that topic. Only seven of the reviews discussed protective mechanisms, although the call to better understand resilient mechanisms is not new (i.e., Rutter, 1993). We will now highlight the resilience mechanisms that have mostly been illustrated. We begin with reviewing the psychological mechanism before referring to biological/neurobiological and genetic resilience mechanisms.

##### Cognitive (Re)appraisal

Empirical evidence consistently shows that cognitive (re)appraisal is beneficial for psychological health (John and Gross, 2004; Garnefski and Kraaij, 2006). The concept relates to traditional ideas of stress and coping models (Lazarus, 1966; Lazarus and Folkman, 1984, 2015). Essentially, our emotional or behavioral reactions in a given situation depend on whether we (re)appraise the situation as harmful or not and whether we have sufficient resources to cope. Central to coping models is the idea of a person as an active agent with control over their life and the ability to clarify the meaning of situations. It seems reasonable to deduce that the individual's ability to regulate or reduce their negative response may offset the risk of developing psychopathological symptoms in stressful or adverse environments. As such, cognitive (re)appraisal plays an important role in resilience research (Kalisch et al., 2015). Troy and Mauss (2011) suggested that cognitive emotion regulation strategies, such as cognitive (re)appraisal or attention control, contribute to resilience. The authors claim that the better an individual is able to regulate their emotions, the more likely they are to be resilient in the face of adversity. Kalisch et al. (2015) even postulate that positive (re)appraisal is one key resilience mechanism. In their Positive Appraisal Style Theory of Resilience (PASTOR), they propose that positive appraisal styles (appraisal, reappraisal, and interference inhibition) constitute a single global mediating resilience mechanism. Further evidence has highlighted that appraisal is linked to the effects of social support on resilience (Mancini and Bonanno, 2009). Generally speaking, the ability to reappraise negative information is considered crucial for developing and maintaining resilience.

##### Cognitive or Regulatory Flexibility

Cognitive or regulatory flexibility was another theme associated with resilience and support to foster a resilient adaptation process (Yao and Hsieh, 2019; Daly, 2020). Flexibility enables individuals to modify cognitive and behavioral strategies to respond accurately to changing environments (Dajani and Uddin, 2015; Whiting et al., 2017). This shift in perspective to a change between different operations is positively associated with resilience (Bonanno and Burton, 2013; Yao and Hsieh, 2019). Supporting evidence comes from studies on resilience that show an association between the individual's cognitive flexibility in their neurochemical stress response and the neural circuitry involved in the stress response (Gabrys et al., 2019). However, to our knowledge, behavioral studies that examine the direct association between cognitive flexibility and resilience are missing (Yao and Hsieh, 2019).

##### Attachment

Rutter (1993) addressed the issue of an underlying mechanism and promoted the importance of secure and supported relations as central to the establishment of self-esteem and self-efficacy. He proposed attachment to be one of four mechanisms, the others being reduction of risk impact, reduction of negative chain reactions, and opening up to opportunities of a positive kind. In fact, most internal factors associated with resilience such as affect regulation, mood repair, and self-esteem and external factors such as stable relationships or family closeness could all be traced back to secure attachment experiences (Atwool, 2006), thus supporting the hypothesis of attachment as an important mechanism to develop resilience. This is evidenced by research showing a link between early attachment experiences, the quality of adult attachment relationships, and global functioning (Holland and Roisman, 2010; Rubinstein et al., 2012). In a recent systematic review and meta-analysis by Darling Rasmussen et al. (2019), the role of attachment as a core feature of the development of resilience was clarified. More specifically, they found attachment to be a pre-requisite of positive adaptation. These bonds did not have to be established within the primary family, but could be extended to teachers, therapists, or other family members.

##### Hardiness

The concept of hardiness was also identified as important to reducing the impact of extreme stress (Bonanno, 2004; Daly, 2020). Hardiness has three dimensions: being committed to finding a purpose in life, having the belief in the power to influence or control surroundings and outcomes, and the belief that both positive and negative experiences promote growing and learning (Kobosa, 1979). For instance, in a qualitative study based on victims of intimate partner violence, hardiness was one of the important themes, in addition to self-efficacy and using social support (Shanthakumari et al., 2014). However, it remained unclear whether hardiness was supposed to be a mechanism or a protective factor.

##### (Neuro)biology

Several structural and functional neural circuitries have been associated with resilience (Yao and Hsieh, 2019). Those brain circuitries include typically cortical-limbic regions like the pre-frontal cortex, the right central execution network, and amygdala (Maren et al., 2013), and mediate a wide range of higher order cognition and emotion regulation. Interestingly the hippocampus was reviewed as an important brain region involved in resilience-related processes like stress or information and emotion processing (Yao and Hsieh, 2019).

##### Genetics

Stainton et al. (2019) reviewed further the involvement of the Differential-Susceptibility Hypothesis as a possible genetic mechanism underlying the resilience mechanism. According to this hypothesis, individuals who carry the vulnerability allele are much more susceptible to both positive and negative contextual influences (Bowes and Jaffee, 2013). For instance, when similarly exposed to adverse contexts, individuals with the protective allele show a reduced risk of developing psychopathology compared to those carrying the vulnerability allele. However, the genes do not function as a protective factor (Kim-Cohen and Turkewitz, 2012). Unless the individual was confronted with adversity, no or only little effects were found (Rutter, 2006).

## Conclusion

Our literature synthesis shows that despite a high attractiveness of the concept and a growing research body, the construct of resilience still lacks clarity in definition, which poses a number of methodological problems in the adult health literature. This discrepancy in the use of the term might impair a unified conceptualization on resilience within and between different scientific fields. Based on our analysis, we discussed three areas regarding the process-oriented approach of resilience we thought worth reconsidering. Firstly, we found conceptual difficulties in the establishment of surrogate outcomes or assessments of resilience. Secondly, although resilience is commonly defined as a process, there is no consensus as to the description of a resilient trajectory. Bonanno and Diminich (2013), Bonanno et al. (2015) argued that some of the confusion between different trajectories emerged because of differences in focus between developmental and adult (trauma) researchers. Whereas, developmental scientists show a greater interest in chronic adversity, adult research is focused on acute life events. It is also worth noting that several other trajectories that are related to psychopathology rather than resilience are found in longitudinal empirical studies: chronic distress, delayed evaluation, continued (pre-)distress, and distress after improvement trajectory, respectively (Bonanno and Diminich, 2013).

Furthermore, despite strong arguments in favor of a prospective process-based approach, many research designs are still retrospective and point-based (Chen and Bonanno, 2020). Although researchers have already begun to disentangle some of the conceptual flaws regarding differences in trajectories based on pre-adverse baseline characteristics like chronicity, socioeconomic factors, or baseline mental health, there is still a lot we do not know. From a conceptual viewpoint, we should be careful about making broad statements on overall resilience given the wide range of operationalization attempts and different methodological approaches that account for many of the differences in the nature of resilience found in the research to date.

Nowadays, research understands resilience as a multifaceted adaptation process that can fluctuate over time and between different types of adversity. Although the conceptualization of resilience has gained some stability as a dynamic multidimensional process-oriented approach over the last two decades, this does not mean that the remaining debate around this concept is obsolete (Stainton et al., 2019). One key to understanding the resilience concept is accurate understanding of the dynamic resilience process (Kleim and Kalisch, 2018), the interaction between internal and environmental protective factors that moderates the negative impact of a crisis (Masten and Motti-Stefanidi, 2020). Within this macro-category, several resources, including (neuro)biological, personal, social, and cultural ones, have been identified. Given the great attention paid to detecting protective outcomes, it is somehow surprising that the underlying mechanisms are still poorly understood. To our knowledge, most of the basic mechanisms such as those Rutter (1993) proposed still lack empirical support or at least have not been addressed in the revealed literature reviews. We highlighted different psychological, (neuro)biological, and genetic mechanisms. Note that this list does not claim to be exhaustive. For instance, Infurna (2020) proposed anticipation of the occurrence of adversity as a possible missing key element in psychological concepts of resilience. Expecting future adversity might foster proactive coping to prevent the adverse event from occurring in the first place or lessen the negative impact upon the individual. He described various scenarios to which anticipation could be applied. One example is related to the research field of the elderly. Here, anticipation might include the preparation for potential loss of independence, a living will or advanced health care directive, or enough financial resources. He argued further that anticipation might also support the process of building up resources or knowledge that decreases vulnerability to later stressors and leads to a steeling effect (Rutter, 2012). Another key mechanism might be how individuals incorporate their experiences into a his/her narrative identity, which is defined as an individual's internalized life story (Infurna and Jayawickreme, 2019). The recent high resonance of narrative-based therapies in trauma research may give this hypothesis a further boost (Lely et al., 2019; Worley et al., 2020). Furthermore, a narrative-based resilience mechanism may represent a link to an interdisciplinary approach of social sciences with the humanities, an area that deserves more attention in light of the call for more interdisciplinary work (Richter, 2017; Lindert et al., 2018). Faye et al. (2018) further review neurobiological mechanisms of resilience in the elderly. To solve the mystery of the resilience concept, it may be important not only to take a closer look at the underlying premises that are present before, during, and after confrontation with a crisis, but also at the baseline characteristics of a given population. Next, we consider how this might be accomplished in future research. We conclude with implication for the clinical context.

### Future Directions

In line with other authors (i.e., Infurna and Luthar, 2018; Kleim and Kalisch, 2018; Infurna, 2020), we highlight several conceptual and methodological directions that are important for future research. Conceptual components discuss how resilience is defined and studied. Methodologically, we propose a greater focus on prospective longitudinal designs with interdisciplinary and international cooperations that combine mixed-methods approaches. We also highlight some clinical implication.

#### Conceptual Considerations

From a conceptual point of view, although there is an increasing consensus about a multidimensional process-oriented definition of resilience, the operationalization of the construct is still unclear and heterogeneous. Firstly, we found that authors used different adaptation criteria to characterize a resilient outcome. Mostly, the presence or absence of health/illness or functionality was used to estimate resilience. However, the multidimensional nature of the process-oriented approach requires consideration of multiple surrogate outcomes for resilience. Until now, a great body of research has focused on measures that relate to a categorical binary one-measure outcome. Instead, several reviews call for implementation and specification of different cross-domain surrogate outcomes for resilience (Kunzler et al., 2018; Infurna, 2020). For instance, Infurna (2020) proposed a broader focus including the individuals' beliefs about themselves, how they view themselves in relationship to others, and their ability to engage with others. A further consideration is to focus on outcomes that are not solely based on self-reports in the form of RSs. For adults, an external assessment based on their partners', children's, friends', or colleagues' opinions of how the individual functions in their different life roles might also be worth considering (Infurna and Luthar, 2018).

Another critical point concerns who determines whether a positive outcome is an appropriate surrogate outcome of resilience. Those are routinely evaluated through the prism of a Western scientific discourse that may lack sensitivity to certain social and cultural criteria (Ungar, 2008; Rudzinski et al., 2017). For instance, a well-accepted criterion like education or employment may not be an attractive goal if it is meaningless in a specific social and cultural environment. The individual in question may remain resilient in their own cultural context (Ungar, 2008). A good example of a Western cultural bias is the aforementioned idea of personal agency. This individualistic view does not take into account the role of social organizations, collective ideologies, or even propaganda (Ratner, 2000). Without considering the importance of differences in cultural agency, it is not surprising that a recent attempt to apply the RS, which is based on individual agency, failed in Africa (Mendenhall and Kim, 2019). The authors proposed “a rethink of medical anthropology's emphasis on the concept of suffering alongside the concept of resilience by cultivating a lens that moves within and between what fosters sickness and wellness” (Mendenhall and Kim, 2019, p. 320). Furthermore, functionality may depend on the individual's personal competencies and preferences. A person may display high life satisfaction despite not reaching goals defined as desirable by a particular society. Moreover, affiliations to religious and spiritual communities or concepts, with their specific conceptions of what constitutes “a good life,” differ enormously, even within Western cultures. For instance, being part of a religious community plays a greater role in America than in Germany (Pollack, 2016). From this viewpoint, it would be of considerable importance to work in international and intercultural scientific groups to diminish such a Western bias.

In the future, more interdisciplinary research is needed (i.e., Richter, 2017; Lindert et al., 2018). From the authors' own interdisciplinary discussions with experts from the humanities, two important aspects that seem underrepresented in the resilience discourse are the integration not only of active coping strategies and positive emotions, but also of more passive aspects of coping, as well as the importance of unpleasant, “negative” emotions (anxiety, frustration; Richter, 2017). Although such forms of emotional reactions are often viewed as pathological, they can also enhance adaptation to extreme adversity (Bonanno, 2004). The literature suggests that appraising, maintaining, or increasing positive and negative emotions are functional when they provide appropriate and context-specific coping responses (Freund and Staudinger, 2015; Mancini, 2015; Troy, 2015). For instance, in some contexts, downregulating negative emotions may jeopardize a person's chances of positive adaptation. In contrast, facing adversity as inevitable, even when this is accompanied by intense negative feelings, may lead a person to comply with necessary actions and/or accept the helpfulness of others (Freund and Staudinger, 2015). Indeed, “the assumption that successful change is dependent on the patient actually experiencing what it is all about is a central element in almost all conceptualizations of psychotherapy. Thus, many possibilities for introducing this important process element have been developed: through experience-activating procedures in the therapeutic situation such as Gestalt techniques, psychodrama, focusing, and so forth […]” (Grawe, 1997, p. 5).

To facilitate comparison between different studies, researchers should further emphasize the specific surrogate outcome they applied. Increased interdisciplinary work could also sharpen and differentiate overlaps between various constructs (i.e., hardiness, optimism, self-esteem) and resilience.

Another critical point concerns the consideration of other important factors such as sociocultural variables, baseline health or illness, and social support. Those pre-event characteristics can influence vulnerability to the exposure of adversity and determine whether an event is seen as potentially threatening. Between-person differences in levels of functioning prior to the onset of adversity might not only be linked to the adverse event itself, but also to the consequences or outcomes that follow (Infurna, 2020). Wheaton (1990) found that prior social circumstances to a life transition could influence whether the transition was evaluated as potentially stressful.

Taken together, future research should focus on developing suitable theoretical frameworks on distinct domains on resilience. To do so, the broad range of resilience research must be disentangled and sorted into different research topics yielding from adversity, vulnerability, protective factors, and outcomes beyond the concepts of health and illness. We now address some methodical considerations of how this might be addressed in adult health research.

#### Methodological Considerations

When resilience is conceptualized as a context-adaptive multidimensional adaptation process, particular challenges emerge for research. Firstly, the question arises as to what extent the complex process of adaptation can be assessed *per se* (Kleim and Kalisch, 2018). One solution is to step away from studying resilience as the end of a complex adaptation process, or more specifically, to study resilient surrogate outcomes or factors after the adaptation process is already completed (Infurna and Luthar, 2018; Kleim and Kalisch, 2018). In this regard, it would be particularly important to assess the progression of the ongoing adaptation process in longitudinal designs at several time points (Bonanno et al., 2015). The longitudinal designs should cover the range from pre- to post-adversity to track the individual trajectories and assess multiple domains of resilience (Lindert et al., 2018). We believe that longitudinal designs might further help supplement resilience mechanisms. Although this proposition is not really new, we still lack longitudinal studies in the general adult population. Most longitudinal approaches are conducted in military personnel or children. However, study outcomes could not be easily compared, as a recent review of four ongoing longitudinal resilience studies revealed. The authors found variable trajectories across age spans and specific to a variety of influencing factors. Such age- and time-specific influences require further clarification (Lindert et al., 2018).

Thereby, it would be particularly important to investigate exposure to crisis, the progression of mental health and functionality, and related (neuro)psychological, biological, and social process within larger multicenter prospective longitudinal studies. The application of a mixed-methods design including not only quantitative measurements, but also qualitative methods, and linking them would allow accounting for subjective changes as well as the quantification of further surrogate outcomes and help us move away from a static-oriented estimation of resilience to a dynamic multifaceted construct (Kleim and Kalisch, 2018). Implementing qualitative methods might also facilitate the disentangling of resilience mechanisms. They might determine how individuals adapt to adversity and reveal additional insights into between-subject variance in adaptation processes (Infurna, 2020). To link all the information that is gathered in parallel, interdisciplinary approaches should be even more considered.

#### Clinical Implications

The importance of conceptual and methodological considerations for future research is even greater given that resilience is a well-accepted concept for health promotion and the prevention of the development of (mental) diseases or illnesses. Clinicians have to be aware that the majority of intervention studies reporting a benefit of a certain resilience training, are not based upon a clear definition of resilience, are performed on a *post-hoc* assessment, rely on self-reports, and the lack of assessment of the relationship of stressor load and mental health. Thus, it makes it difficult to conclude if the resilience training led to a resilient outcome or not (Chmitorz et al., 2018). Furthermore, clinicians need to keep in mind, that fostering specific protective factors might not be sufficient if the adaptation process itself is not taken into account. Baseline characteristics, individual goal preferences, the social environment, individual resources as well as the crises itself might need to be considered on an individual and contextual level.

### Limitations

In general, it should be noted, that though we aimed to synthesize consistencies on the topic of resilience in adult mental health science, the nature of this article is to some extent narrative and must be read with a certain caution due to a subjective character.

One limitation of this article is the restriction of the database to published reviews within the last 5 years. While we tried to reflect the current scientific discourse on resilience, we may have missed important conceptualizations that have been published recently. We did not include other specific resilience-like constructs such as hardiness. Fletcher and Sarkar (2013), however, showed that those constructs did not reflect the complexity of resilience itself. In addition to the general term “resilience,” researchers sometimes add a number of modifiers to specify a particular context form such as family resilience and (neuro)biological resilience. In the interest of making direct comparisons, however, only reviews that dealt with the conceptualization of the term “resilience” as a standalone construct were included. Consequently, this search strategy may have prevented the inclusion of other forms of resilience. Secondly, we focused only on methodological and conceptual difficulties regarding resilience and its dynamics. An overview of the huge body of empirical results regarding resilience would have exceeded the scope of this article. It is also important to note that reviews included in this article showed several years in the search period dating back until 1,806. Although some of the inconsistencies linked to the term might have already been solved, we believe that most of the highlighted points are still poorly understood in mental health research. Thirdly, we excluded the topic of intervention from our synthesis, which might have contributed a different perspective on resilience. However, we believe it is important to first understand the concept itself before deriving intervention strategies.

Finally, we focused on resilience in adult mental health science, thereby excluding nuances of resilience conceptualizations used in other scientific domains. Our primary concern was to highlight the reasons such an intuitive and attractive concept is so controversial and to facilitate further theoretical and practical work in the field without abolishing the concept itself. We believe that simply exchanging the term “resilience” with a synonym before addressing the aforementioned conceptual and methodological flaws would merely transfer these flaws onto the new term. Similarly, the same concerns apply equally for adversity or crisis, which we have not discussed here, but which are conceptually intertwined with resilience. The concept of resilience remains a construct with very high intuitive appeal and exciting opportunities in research. It deserves more intensive interdisciplinary scientific development.

## Author Contributions

NH wrote the manuscript. MR, KM, FP, LR, and FG provided critical revision of the manuscript for important intellectual content. All authors contributed to the article and approved the submitted version.

## Conflict of Interest

The authors declare that the research was conducted in the absence of any commercial or financial relationships that could be construed as a potential conflict of interest.
